# Memory and Learning Deficits Are Associated With Ca^2+^ Dyshomeostasis in Normal Aging

**DOI:** 10.3389/fnagi.2020.00224

**Published:** 2020-07-16

**Authors:** Arkady Uryash, Valentina Flores, Jose A. Adams, Paul D. Allen, Jose R. Lopez

**Affiliations:** ^1^Division of Neonatology, Mount Sinai Medical Center, Miami, FL, United States; ^2^Department of Research, Mount Sinai Medical Center, Miami, FL, United States; ^3^Malignant Hyperthermia Investigation Unit, St James’ University Hospital, University of Leeds, Leeds, United Kingdom

**Keywords:** neurons, calcium, aging, memory deficits, dantrolene, TRPC

## Abstract

Neuronal intracellular Ca^2+^ homeostasis is critical to the normal physiological functions of neurons and neuronal Ca^2+^ dyshomeostasis has been associated with the age-related decline of cognitive functions. Accumulated evidence indicates that the underlying mechanism for this is that abnormal intracellular Ca^2+^ levels stimulate the dysregulation of intracellular signaling, which subsequently induces neuronal cell death. We examined intracellular Ca^2+^ homeostasis in cortical (*in vivo)* and hippocampal (*in vitro)* neurons from young (3-months), middle-age (12-months), and aged (24-months) wild type C57BL6J mice. We found a progressive age-related elevation of intracellular resting calcium ([Ca^2+^]_r_) in cortical (*in vivo*) and hippocampal (*in vitro*) neurons associated with increased hippocampal neuronal calpain activity and reduced cell viability. *In vitro*, removal of extracellular Ca^2+^ or treatment with SAR7334 or dantrolene reduced [Ca^2+^]_r_ in all age groups and dantrolene treatment lowered calpain activity and increased cell viability. *In vivo*, both middle-aged and aged mice showed cognitive deficits compared to young mice, which improved after dantrolene treatment. These findings support the hypothesis that intracellular Ca^2+^ dyshomeostasis is a major mechanism underlying the cognitive deficits seen in both normal aging and degenerative neurologic diseases.

## Introduction

Perturbations of intracellular Ca^2+^ concentration underlie the increased vulnerability of neurons to age-related processes like cognitive decline and degenerative neurological diseases including, Alzheimer’s and Parkinson’s disease (Disterhoft et al., 1994; Kirischuk and Verkhratsky, 1996; Lopez et al., 2008; Alzheimer’s Association Calcium Hypothesis, 2017; Kumar, 2020). The age-related cognitive decline includes impairment in retrieving intermediate-term memories, especially episodic memories that rely on spatial and temporal contexts (Tromp et al., 2015; Gollan and Goldrick, 2019). Emerging evidence supports the idea that a disruption of the mechanisms that normally regulate intracellular neuronal intracellular [Ca^2+^] plays a critical role in many of the neural dysfunctions underlying chronic brain disorders in aging (Thibault et al., 2007). Furthermore, the Ca^2+^ hypothesis states that intracellular Ca^2+^ dysfunction serves as a precursor and driver not only to aging-associated decrements in neuronal performance but also to the molecular mechanisms underlying neuronal degeneration.

Intracellular resting Ca^2+^ concentration ([Ca^2+^]_r_) is maintained in neurons as a balance of the Ca^2+^ leak from the ryanodine receptor, the extrusion elicited by the plasma membrane Ca^2+^ pump, and sodium/calcium exchanger and plasmalemmal Ca^2+^ entry through the voltage-gated Ca^2+^ channels, transient receptor potential canonical (TRPC) channels, and the store-operated Ca^2+^ entry channels (Berridge et al., 2000; Eltit et al., 2010; Hill and Olson, 2012; Schwaller, 2012);(Wu et al., 2002; Tonkikh et al., 2006). For more than 35 years, evidence has been accumulating that brain aging is associated with dysregulation of intracellular calcium in neurons (Gibson and Peterson, 1987). Age-associated disruption in intracellular Ca^2+^ homeostasis has been related to several mechanisms that include enhanced L-type Ca^2+^ currents (Landfield, 1996; Thibault and Landfield, 1996), a decline in SERCA-mediated Ca^2+^ uptake by the endoplasmic reticulum (ER), oxidative modification of the inositol trisphosphate and ryanodine receptors (Kumar et al., 2018), a decreased expression of FKBP1b (Gant et al., 2015), and increased activity of calpains (Hinman et al., 2004), all of which might lead to an intracellular Ca^2+^ overload, protein degradation and neuronal death (Hinman et al., 2004).

However, the mechanisms governing this neuronal intracellular Ca^2+^ dysfunction and its potential link to cognitive impairment in aging are not fully understood, and consequently, this pathologic mechanism is not currently being targeted as a means of preventing or repairing cognitive dysfunction. Also, some of the potential pathways that could be involved in aging-mediated changes in intracellular [Ca^2+^] have not been fully studied. One of those Ca^2+^ pathways is the Ca^2+^ influx mediated by the transient receptor potential canonical (TRPC) channels (Liguori et al., 2018; Maria-Ferreira et al., 2020). TRPC proteins comprise nonselective cation channels in the sarcolemma that permit the permeability of Ca^2+^ and Na^+^ into the cells (Nilius and Szallasi, 2014). TRPC channels have been implicated in diverse neuronal physiological functions, but have also been linked with neurodegenerative processes (Bezprozvanny, 2009; Takada et al., 2013; Sukumaran et al., 2017; Maria-Ferreira et al., 2020). Another possible mechanism for increased intracellular [Ca^2+^] that may be involved in an increase in Ca^2+^ leak from the ER *via* ryanodine receptors (RyR; Lacampagne et al., 2017). Three mammalian isoforms of the RyR receptors have been described: RyR1, RyR2, and RyR3. Although all three isoforms can be found in the brain, the RyR2 isoform is the predominant isoform expressed in the cerebral cortex and the hippocampus (Giannini et al., 1995). An enhanced RyR Ca^2+^ leak has been postulated as contributing to Alzheimer pathogenesis, and pharmacological intervention to modify the leak normalized cognitive functions (Peng et al., 2012; Liang and Wei, 2015; Lacampagne et al., 2017).

The present study aimed to explore the involvement of ER Ca^2+^ leak *via* RyR receptors and the Ca^2+^ influx mediated by the TRPC channels on the resting intracellular Ca^2+^ dysregulation observed with aging. We hypothesized that: (i) aging is associated with a progressive increase in resting intracellular Ca^2+^ concentration ([Ca^2+^]_r_) and Na^+^ ([Na^+^]_r_); and (ii) pharmacological modification of either the ER-RyR1 leak or the Ca^2+^ influx mediated by TRPCs channels will reduce resting [Ca^2+^]_r_ and [Na^+^]_r_ and revert aging-related cognitive deficits.

## Materials and Methods

### Animals

Male and female 3–4-months (Young), 12–14-months (Middle Age), and 24–26-months (Aged) C57BL6J mice obtained from breeding colonies at the Mount Sinai Medical Center, from founders initially obtained from the Jackson Laboratory (Bar Harbor, ME, USA) were used as experimental models. The three mice ages: young, middle age, and aged were selected to be the equivalent to approximately 10, 40, and 80 human years based on the fact that one human year is equivalent to nine mice days (Dutta and Sengupta, 2016). Despite the vast differences in their lifespan, humans and mice show similar molecular mechanisms of aging (Demetrius, 2006). Mice were accommodated four per cage with food and water available ad libitum and were maintained on a 12 h light/dark cycle. All protocols used in the study were performed following the recommendations in the Guide for the Care and Use of Laboratory Animals of the National Institutes of Health and were approved by the institutional IACUCs were the animal experiments were performed.

### Anesthesia

For *in vivo* experiments mice fasted (6 h), and water was withheld for 1 h before anesthesia. Mice were anesthetized with ketamine/xylazine (100/5 mg/kg body weight) and then placed on a temperature-controlled pad. Its nose was inserted into an anesthesia mask (Kent Scientific, Torrington, CT, USA) and all mice inhaled ultra-pure grade air (Airgas, Miami, FL, USA) and were allowed to breathe spontaneously. The vital signs (respiratory rate, heart rate, oxygen saturation, and rectal temperature) were monitored throughout the procedure. Tail and/or toe pinches were used to ensure the animal was fully anesthetized throughout the investigative procedures. Body temperatures were maintained between 36.9 and 37.3°C using a Peltier thermostatic low noise temperature controller ATC2000 (WPI, Sarasota, FL, USA) with feedback control from a rectal probe.

### Surgical Procedures

Anesthetized mice were placed in a stereotaxic frame, and the head was firmly secured with the ear bars. Eye ointment was applied to prevent the animal’s eye from drying out. Dexamethasone (0.2 mg/Kg) was administered subcutaneously to prevent brain swelling. Pilot experiments show that dexamethasone at the dose used in the present study does not modify neuronal [Ca^2+^]_r_ (data not shown). After shaving and disinfection of the surgical site, the skin above the frontal-parietal temporal lobes was removed, and a mixture of lidocaine and epinephrine solution was injected into the periosteum to avoid bleeding and to reduce pain. A small circle of about 1 cm in diameter etched away with the aid of a pneumatic dental drill. The drilling was stopped when a very thin layer of bone was left. By pushing gently in the center of the draw circle, and the aid of small forceps, the remaining bone was removed from the skull. Gelfoam sponge was used in case of bleeding. Petrolatum was deposited around the craniotomy to create a “chamber,” which was filled and continuously perfused at a rate of 3–5 ml/min with warm sterile artificial cerebrospinal fluid (ACSF) equilibrated with a 95/5/% O_2_/CO_2_ mixture.

### Ca^2+^-Selective Microelectrodes

Double-barreled Ca^2+^-selective microelectrodes were prepared and individually calibrated as described previously (Eltit et al., 2013). Ca^2+^ ionophore II (ETH 129; Fluka Sigma–Aldrich, St. Louis, MO, USA) was used to backfill the Ca^2+^-selective microelectrode. Resting membrane potential (Vm) and Ca^2+^ potentials were recorded *via* a high impedance amplifier (FD-223-WPI, Sarasota, FL, USA), as described previously (Lopez et al., 2018b). After obtaining measurements of resting [Ca^2+^]_r_, all microelectrodes were recalibrated. If the initial and final calibration curves did not agree within 3 mV, data from that microelectrode were discarded.

### Measurements and Recording of [Ca^2+^]_r_ *in vivo*

The pre-calibrated Ca^2+^-selective microelectrode was gently lowered to make contact with the artificial cerebrospinal fluid that filled the petrolatum chamber around the craniotomy using a three-axis micromanipulator. The microelectrode was then repositioned under direct visualization with the stereomicroscope. Neuron impalement was carried out under “blind conditions.” We were able to differentiate neurons from glial cells and astrocytes based on their resting membrane potential (Vm). Glial cells and astrocytes had a resting Vm in the vicinity of −58 mV while intact healthy neurons have a more polarized Vm which is equal to or more negative than −65 mV. Criteria for successful impalement included an abrupt drop to a steady level of Vm equal to or more negative than −65 mV, a stable recording of both Vm and Ca^2+^ potential for more than 1 min and an abrupt return to baseline on the exit of the microelectrode from the cell. Since encountering a cortical neuron was a random process, multiple attempts were conducted until a neuron that met the above criteria was found and remained quiescent (no spontaneous firing) during the recording. If the neuron was not quiescent the electrode was removed, and an alternative neuron found for the recording.

### Preparation of Primary Hippocampal Pyramidal Neurons

Young, middle-aged, and aged mice were euthanized by cervical dislocation, and primary hippocampal pyramidal neurons were prepared as previously described (Robin et al., 2017). It is well established that most of the cognitive process depends upon proper hippocampal function, and that hippocampal function appears to be particularly age-sensitive(Foster, 1999). All experiments were conducted on neurons cultured for 7 days at 37°C, 5% CO_2_, and room air.

### Measurements and Recording of [Ca^2+^]_r_ and [Na^+^]_r_ *in vitro*

Resting intracellular Ca^2+^ and Na^+^ concentrations ([Ca^2+^]_r_ and [Na^+^]_r_**)** were measured in isolated hippocampal pyramidal neurons from young, middle-aged, and aged mice using double/barreled Ca^2+^-and Na^+^-selective microelectrodes as previously described (Lopez et al., 2005; Eltit et al., 2013; Robin et al., 2017). Vm, Ca^2+^, and Na^+^ potentials were recorded *via* a high impedance amplifier (WPI FD-223; Word Precision Instruments, Sarasota, FL, USA) as described previously (Lopez et al., 2018b). All experiments were carried out at 37°C.

### Calpain Activity

Calpain I and II activity were assessed in hippocampal neurons isolated from young, middle-aged, and aged mice using the Calpain-Glo protease assay (Promega, Madison, WI, USA). Neurons were incubated with the Calpain-Glo reagent for 10 min at 37°C, and luminescence was measured using a Synergy 2 plate reader (BioTek, Winooski, VT, USA). Intensity values for middle-aged and aged neurons are reported normalized to values from young neurons.

### Neuronal Viability

Viability was determined in primary hippocampal neuron cultures using the 3-(4,5-dimethylthiazol-2-yl)-2, 5-diphenyltetrazolium bromide (MTT) assay according to the manufacturer’s protocol. After washing, neurons were incubated with 0.5 mg/ml MTT (Sigma–Aldrich, St. Louis, MO, USA) solution at 37 °C for 4 h. The medium was removed, and then 150 μl DMSO was added to each well and mixed thoroughly to dissolve the generated formazan. Data collected from young, middle-aged, and aged mice are represented as a reduction in MTT concentration relative to neurons from young mice.

### Morris Water Maze

Cognitive function was determined using the Morris water maze (MWM) test (Morris, 1984; Sesay et al., 1996; Vaillend et al., 2004; Handattu et al., 2009; Faes et al., 2010; Lopez et al., 2018b). Young, middle-aged, and aged mice received three trials per day, with an inter-session interval of 30 min for four consecutive training days. After every trial, each mouse was towel-dried and kept warm with a heat lamp before being returned to its home cage, where it had free access to food and water. Mice were allowed to swim for 80 s or until the platform was reached. In the case where the mouse did not find the platform, it was guided to the platform and allow to stay there for a short period. Upon completion of the 4 days training period (TP) on day 5, the platform was removed to allow for probe testing. Three parameters were measured: (a) the escape latency time (ELT; time taken by each mouse to reach the site where the platform had been located), which was used as an index of acquisition or learning. The less time it took a mice to reach the site where the platform had been located, the better the learning ability; (b) the time spent by the mice in the target quadrant (TTQ)—in which was the platform previously located; and (c) the number of times the mouse crossed over the area where the platform was previously hidden (NETQ). The experiments were conducted in the morning between 9 am and 11 am. The behavior of the mice in the pool was recorded by a video tracking system (WatermazeScan, Reston, VA, USA) and stored for later analysis.

The Morris learning test was conducted in young, middle-aged and aged mice, which were divided randomly into two groups: Group 1: *Testing the effect of aging on spatial learning and memory*. Young (*n*_mice_ = 5), middle-aged (*n*_mice_ = 5), and aged mice (*n*_mice_ = 5) did not receive any treatment before or during the TP (TP) and/or probe trial (PT); Group 2: *Testing the effect of dantrolene on cognitive abilities*. All mice received dantrolene (DANT; 1 mg/kg, i.p.) for 5 days. before and every day during the TP and PT periods (*n*_mice_ = 5 mice per age group). The dantrolene dose used (1 mg/kg, i.p.) was chosen based on the fact that in preliminary studies we were able to show that this dose significantly reduced neuronal [Ca^2+^]_r_ but did not interfere with the ability of the mice to swim (unpublished observations).

### Solutions

The sterile artificial cerebrospinal fluid (ACSF) used for *in vivo* studies contained in (mM) 135 NaCl, 1.8 KCl, 26 NaHCO_3_, 1.25 NaH_2_PO_4_, 2 CaCl_2_, 1 MgCl_2_, and 5 glucose (pH 7.4; when bubbled with 95% O_2_ and 5% CO_2_). Ringer-Locke’s solution used for *in vitro* studies contained the following (in mM): 135 NaCl, 5 KCl, 2 CaCl_2_, 1 MgCl_2_, 5 glucose, tetrodotoxin (TTX 1.5 μM), 3.6 NaHCO_3_ (pH 7.4). Ca^2+^ free solution was prepared by omitting Ca^2+^ and adding 1 mM EGTA. In all experiments, ACSF and the Ringer-Locke’s solution were aerated with a mixture of 95% O_2_ and 5% CO_2_. Dantrolene solution was prepared by dissolving the dantrolene powder in dimethyl sulfoxide and then adding the appropriate amount of dissolved dantrolene to the Ringer-Locke’s solution to make the desired concentration (20 μM). SAR7334 solutions (Tocris, Minneapolis, MN, USA) were prepared by adding concentrated stocks of the drug to the Ringer-Locke’s solution to a final concentration of 0.1 or 1 μM. We added TTX (1.5 μM; Abcam, Cambridge, MA, USA) to the Ringer-Locke’s solution to suppress Ca^2+^ oscillations because spontaneous Ca^2+^ oscillations interfered with the measurement of [Ca^2+^]_r_. In previous experiments, we have demonstrated that TXX did not alter either the resting membrane potential or [Ca^2+^]_r_ in hippocampal neurons that were not firing spontaneously (Robin et al., 2017).

### Statistical Analysis

Values are expressed as mean ± SD. The data were subjected to analysis of variance (one-way ANOVA) followed by Tukey’s *post hoc* comparisons tests to determine significance. *p* < 0.05 was considered as statistically significant. Analysis of these data failed to show sex differences; therefore, the data from male and female mice were combined for analysis. *n_mice_*: indicates the number of mice used and *n_cells_*: represents the number of successful measurements.

## Results

### Effect of Aging and Dantrolene on [Ca^2+^]_r_ in Cortical Neurons *in vivo*

**Figure 1:**
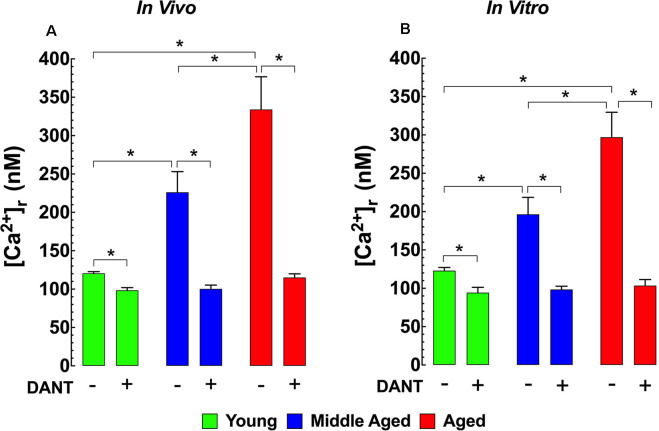
Increased [Ca^2+^]_r_ in aged neurons. Effect of dantrolene. [Ca^2+^]_r_ was measured in quiescent young, middle-aged, and aged cortical neurons *in vivo* **(A)** and hippocampal pyramidal neurons *in vitro* **(B)** using double-barreled ion-specific microelectrodes. [Ca^2+^]_r_ was significantly higher in the middle-aged and aged than young neurons in both conditions. Dantrolene (2 mg/Kg IP) *in vivo* **(A)** or (20 μM) *in vitro* **(B)** reduced [Ca^2+^]_r_ in all age-groups. The reduction of [Ca^2+^]_r_ greater in aged than young neurons. On the horizontal axis are indicated the experimental conditions used to measure [Ca^2+^]_r_ *in vivo* [Ca^2+^]_r_ measurements*: n*_mice_ = 3–5/age group*, n_cell_* = 8–12/age group; *in vitro* [Ca^2+^]_r_ measurements: *n*_mice_ = 4–5/ per age group*, n_cell_* = 11–17/age group. Values are expressed as means ± S.D. One-way ANOVA followed by Tukey’s *post hoc* comparisons, *p ≤ 0.05.

The impairment of intracellular Ca^2+^ homeostasis is considered to be a key pathological factor leading to aging-related neuronal dysfunction (Raza et al., 2007). Therefore, we measured [Ca^2+^]_r_ *in vivo* in polarized cortical neurons from young (3-months), middle-aged (12-months), and aged (24-months) mice to determine if there were any changes associated with normal aging in mice. In young mice, cortical neuronal [Ca^2+^]_r_ was 121 ± 2 nM. Interestingly in middle-aged WT mice neuronal [Ca^2+^]_r_, increased 1.9-fold to 226 ± 27 nM, and in aged mice, it rose 2.7-fold to 334 ± 43 nM (Figure 1A). Dantrolene has been previously shown to reduce [Ca^2+^]_r_ in skeletal muscle and neurons (Lopez et al., 1987a,b; Robin et al., 2017). We found that pre-treatment of mice with Dantrolene (2 mg/kg IP) 60 min before measurements, decreased cortical neuronal [Ca^2+^]_r_ in all age-groups. However, the reduction of [Ca^2+^]_r_ in the middle-aged and aged cortical neurons was greater than in young neurons reaching levels that were the same or similar to those measured after dantrolene treatment in young mice (Figure 1A).

### Effect of Aging and Dantrolene on [Ca^2+^]_r_ in Hippocampal Neurons *in vitro*

To allow a more mechanistic study of potential neuronal [Ca^2+^]_r_ dyshomeostasis in aging WT mice, we carried out measurements of [Ca^2+^]_r_ in isolated, polarized hippocampal pyramidal neurons from all three age groups. Similar to our *in vivo* findings from cortical neurons, day 7 cultured hippocampal neurons from middle-aged mice have a 1.7-fold higher [Ca^2+^]_r_ (199 ± 23 nM) and aged mice have a 2.4-fold higher [Ca^2+^]_r_ (286 ± 40 nM) than neurons from young mice (120 ± 2 nM; Figure 1B). Similar to our results in cortical neurons *in vivo*, when day 7 cultured hippocampal neurons were exposed to 20 μM dantrolene 15 min before recording [Ca^2+^]_r_ it reduced [Ca^2+^]_r_ all three age groups to levels that were below those in untreated young neurons (Figure 1B).

### Increased Hippocampal Neuronal [Ca^2+^]_r_ is Mediated in Part by TRPC Channels

**Figure 2:**
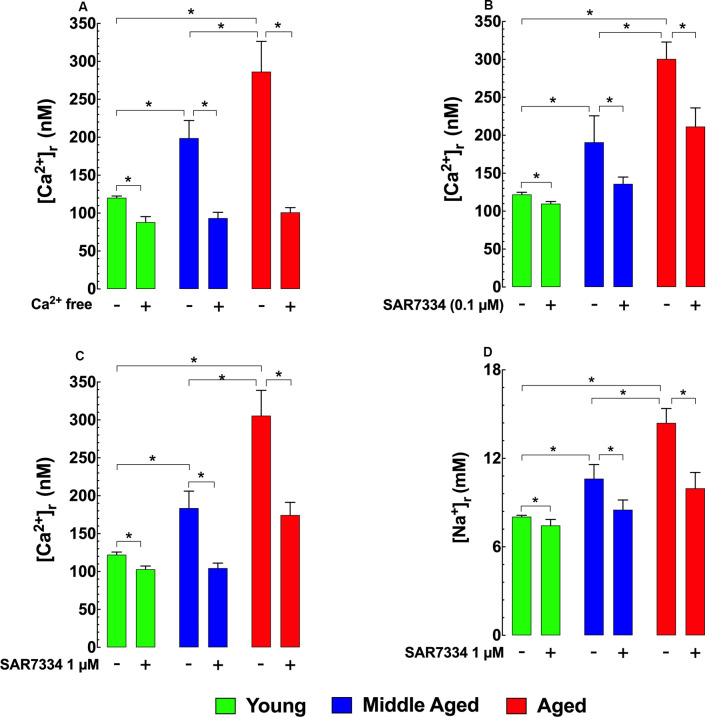
Effects of Ca^2+^ free solution and SAR7334 on [Ca^2+^]_r_ and [Na^+^]_r_ in hippocampal pyramidal neurons. Panel **(A)** shows that removal of extracellular Ca^2+^ significantly lowered the [Ca^2+^]_r_ in all age-group; however, the magnitude of the reduction of [Ca^2+^]_r_ was more evident in aged than young neurons. Panels **(B,C)** illustrate SAR7334 elicited a dose-dependent reduction of [Ca^2+^]_r_ in the young, middle-aged and aged neurons, being more marked in aged neurons. Panel **(D)** exemplifies the reduction provoked by SAR7334 on [Na^+^]_r_, which was greater in aged than young neurons. On the horizontal axis are indicated the experimental conditions used to measure [Ca^2+^]_r_ and [Na^+^]_r_. For [Ca^2+^]_i_ measurements*: n_mice_* = 3–5/age group, *n_cell_* = 9–16/age group. For [Na^+^]_i_ measurements*: n_mice_* = 4/per age group, *n_cell_* = 12–15/age group. Values are expressed as means ± S.D. for each condition. One-way ANOVA followed by Tukey’s *post hoc* comparisons, *p ≤ 0.05.

To determine how [Ca^2+^]_r_ is affected by extracellular sources of Ca^2+^, we incubated hippocampal neurons in Ca^2+^ free media for 15 min and found that this treatment reduced [Ca^2+^]_r_ in the two older age groups to levels similar to those in young neurons (Figure 2A). To determine the potential contributions TRPC3 and TRPC6 channels as a possible mechanism for the apparent increased extracellular Ca^2+^ entry observed in middle-aged and aged hippocampal neurons we incubated young, middle-age, and aged neurons with the TRPC3/TRPC6 channels blocker SAR7334 (Maier et al., 2015). SAR7334 reduced [Ca^2+^]_r_ in all age groups in a dose-dependent manner; however, the effect of SAR7334 was greater with increasing age (Figures 2B,C). Because TRPC channels are nonselective cation channels Sawamura et al., 2017; Samanta et al., 2018 and their increased activity might also allow significant Na^+^ entry, we also measured intracellular Na^+^ concentration ([Na^+^]_r_). We found that [Na^+^]_r_ was 1.3-fold higher (10.6 ± 0.9 mM) in middle-aged neurons), and 1.8-fold higher in aged neurons (14.4 ± 0.9 mM) than in young neurons (8.0 ± 0.1 mM; Figure 2D). As was the case for [Ca^2+^]_r_, SAR7334 (1 μM) significantly reduced [Na^+^]_r_ in neurons of all age groups, and like its effect on [Ca^2+^]_r_, the reduction was more prominent in middle-aged (20%) and aged neurons (31%), compared to young neurons (6%) (Figure 2D).

### Dantrolene Treatment Decreases Calpain Activity and Increases Hippocampal Cell Viability

**Figure 3:**
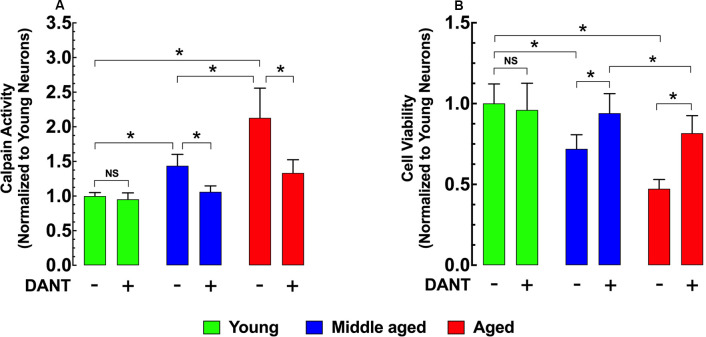
Effects of dantrolene on calpain activity and cell viability in aged hippocampal neurons. Calpain activity was higher in middle-aged and aged hippocampal neurons than young neurons **(A)**, and these differences were accompanied by a decreased cell viability in middle-aged and aged hippocampal neurons compared to young neurons **(B)**. Dantrolene (20 μM) reduced calpain activity, and increased cell viability in aged hippocampal neurons. On the horizontal axis are indicated the experimental conditions. For calpain: *n_mice_* = 3/age group; for cell viability: *n_mice_* = 4/per age group. Values are expressed as means ± S.D. for each condition. One-way ANOVA followed by Tukey’s *post hoc* comparisons, *p ≤ 0.05; NS, not significant.

Intracellular Ca^2+^ overload plays a major role in the development of cell injury (Cross et al., 2010). Activation of the Ca^2+^-dependent protease calpain is believed to be one mechanism by which increased [Ca^2+^]_r_ can cause damage (Hosfield et al., 1999; Cheng et al., 2018). Compared to young hippocampal neurons, calpain activity was significantly elevated in both middle-age (43%) and aged (113%) neurons (Figure 3A). Reducing neuronal [Ca^2+^]_r_ with 30 min of dantrolene (20 μM) pretreatment decreased the calpain activity by 26% in middle-aged and 37% in aged neurons compared to untreated controls (Figure 3A). In a direct measurement of hippocampal neuronal viability using the MMT assay, we found that the increased calpain activity in middle-aged and aged neurons was associated with a 28% and 53% decrease in viability respectively than young neurons (Figure 3B) and that the decrease of calpain with pretreatment with dantrolene was accompanied by a significant increase in neuronal viability, 31%, and 73%, respectively in middle-aged and aged neurons compared to untreated neurons (Figure 3B).

### Cognitive Impairment in Aged Mice and the Ability of Dantrolene to Attenuate Memory Deficits

**Figure 4:**
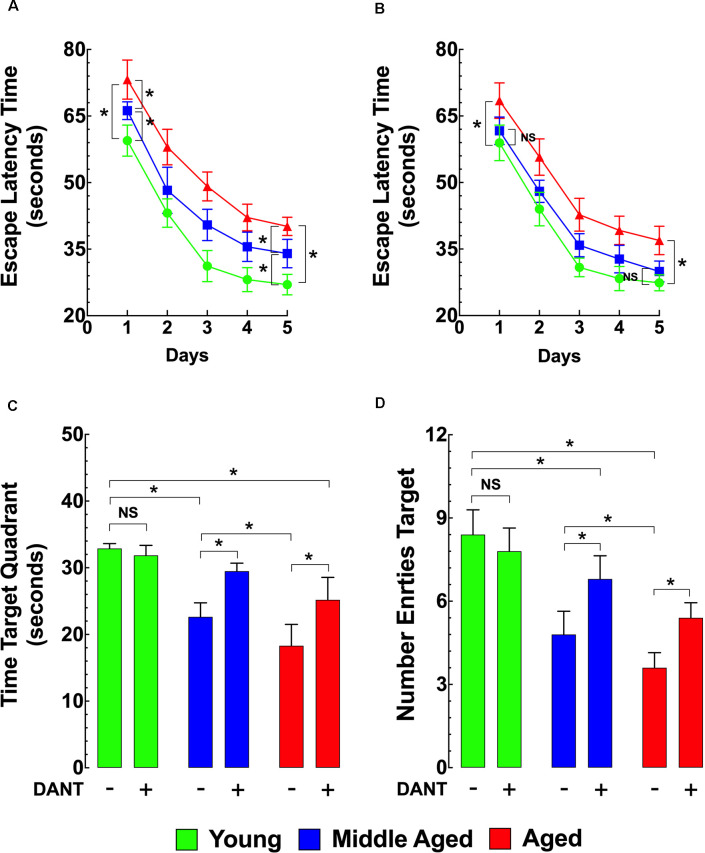
Effects of aging on cognitive function. Effects of dantrolene. The Morris water maze (MWM) was used to assess spatial learning and memory in young, middle-aged, and aged mice. Middle-aged and aged mice showed a reduced escape latency time (ELT; **A**), a time in the target quadrant **(C)**, and the number of entries into the target quadrant **(D)** compared to young mice. Dantrolene (1 mg/kg, i.p) for 5 days. before and during the training period (TP), and probe trial (PT) periods improved all of the parameters studied **(B–D)**. On the horizontal axis of panels **(C,D)** are indicated the experimental conditions. Values are expressed as mean ± SEM from *n_mice_* = 10 per age group. One-way ANOVA followed by Tukey’s *post hoc* comparisons, *p ≤ 0.05; NS, not significant.

As shown in Figure 4A, using the MWM as the method of assessing memory and learning, the ELT for middle-aged and aged mice was significantly longer compared to young mice during the whole TP and on the PT day. Dantrolene treatment (1 mg/kg/day, i.p.) for 5 days. before and during the TP and PT periods (10 days of treatment) significantly improved the escape latency in middle-aged and aged mice but had no significant effect in young mice (Figure 4B). In middle-aged mice, after the dantrolene treatment, the ELT values were not significantly different compared to young mice during the PT period (Figure 4B). However, in the aged group after the dantrolene treatment, the ELT values were significantly different (at any point during the PT period) compared to young mice. In assessments of memory retention, evaluated by the time spent in the quadrant and the number of times the mouse crossed over the area where the platform was previously hidden determined by the number of entries into the quadrant, both parameters showed an age-related deficit in untreated middle-aged and aged mice compared to young mice and dantrolene treatment significantly improved both of these deficits (Figures 4C,D).

## Discussion

This is the first study to directly examine changes in intracellular Ca^2+^ homeostasis using intracellular Ca^2+^ selective microelectrodes—*in vivo*—in cortical neurons and—*in vitro*—in hippocampal neurons associated with normal aging in WT mice. Mice were studied at three different ages, selected to be the equivalent to approximately 10 (young), 40 (middle-aged), and 80-year-old (aged) humans.

In young mice, neuronal intracellular [Ca^2+^] is maintained in the range of 100–120 nM in resting cells against a large extracellular concentration gradient (Lopez et al., 2008; Robin et al., 2017; Lopez et al., 2018a). Neuronal [Ca^2+^]_r_ is regulated by multifaceted mechanisms that balance Ca^2+^ influx and release from intracellular stores with intracellular sequestration and extracellular extrusion (Brini et al., 2014). Perturbations of any of the mechanisms involved in intracellular Ca^2+^ regulation will ultimately lead to a sustained rise of neuronal [Ca^2+^]_r_. Several Ca^2+^-dependent regulatory processes undergo age-dependent changes, which correlate with cognitive decline (Gant et al., 2006; Bodhinathan et al., 2010); (Disterhoft et al., 1996). Also, studies have shown that reversal of the intracellular Ca^2+^ dyshomeostasis using Ca^2+^ channel antagonists (Disterhoft and Oh, 2006), antioxidants (Cartford et al., 2002), FKBP1b overexpression (Gant et al., 2015) and Ca^2+^ chelating agents as BAPTA (Tonkikh et al., 2006) improves aged-dependent cognoscitive deficits. We demonstrated *in vivo* that [Ca^2+^]_r_ in cortical neurons of middle-aged and aged mice is chronically elevated compared with young mice and that treatment with dantrolene can reduce [Ca^2+^]_r_ in middle aged and aged mice to levels near those of treated young mice. Similarly, [Ca^2+^]_r_ in isolated hippocampal pyramidal neurons after 7 days in culture show the same intracellular Ca^2+^ disturbances in middle-aged and aged neurons *in vitro* that we measured in cortical neurons *in vivo* and in these studies we were able to show that the changes in [Ca^2+^]_r_ were accompanied by a parallel increases in [Na^+^]_r._ Like the *in vivo* experiments, treatment of cultured neurons with dantrolene lowers [Ca^2+^]_r_ in all age groups. Further research must be carried out to explore more in-depth the role of intracellular Ca^2+^ dysregulation observed with aging.

One important finding in this study is that our data show that intracellular Ca^2+^ dyshomeostasis occurs in mice at younger ages (12 months old) than those which are usually considered aged (24 months or older). This has important clinical implications since it demonstrates that alterations of neuronal [Ca^2+^]_r_ are progressive with age and begin at a much earlier age than has been previously assumed. Therefore, to be effective over the long term, therapies oriented to prevent cognitive decline might need to be initiated at a much earlier age. Our data showing an age-related increase in [Ca^2+^]_r_ with age are in agreement with the findings reported by Tonkikh et al. (2006) using hippocampal slices, and by Raza et al. (2007) and Hajieva et al. (2009) in hippocampal neurons who found that [Ca^2+^]_r_, as determined by fluorescent dyes was elevated in aged (24 months old or older) neurons compared to young animals. However, other reports (Thibault et al., 2001; Lopez et al., 2008) found no differences in [Ca^2+^]_r_ between young and aged or middle-aged neurons. Diverse factors could account for this disagreement. Among them are differences in the methods used to isolate the neuronal preparations, the Ca^2+^ indicator employed to carry out the intracellular measurements, the age and type of neurons studied, neuronal viability status of the model studied, and the genetic strain of the animals. Our *in vivo* and *in vitro* studies were carried out in cortical and hippocampal pyramidal neurons that were proven viable because they had proven normal membrane potentials thus avoiding the potential for having included injured cells in our experimental groups. Additionally, [Ca^2+^]_r_ was measured directly with Ca^2+^ selective microelectrodes, to overcome the limitations imposed by the currently available fluorescent and metallochromic Ca^2+^ indicators (Alvarez-Leefmans et al., 1981; Hove-Madsen and Bers, 1992).

### Ryanodine Receptor Leak and Neuronal Ca^2+^ Dysfunction

At normal physiological conditions, a fraction of ER Ca^2+^ leak occurs as uncoordinated openings of individual RyRs. This leak component can serve as an important protective mechanism against the ER Ca^2+^ overload in excitable tissues (Zima et al., 2010). However, changes in neuronal redox state, such as the increased oxidative condition exhibited by aged neurons, affect the resting RyR-leak and the RyR-mediated Ca^2+^ release (Paula-Lima et al., 2014), since RyR channels are highly redox-sensitive (Hidalgo and Donoso, 2008). ER depletion has been associated with activation of Ca^2+^ entry through store-operated Ca^2+^ channels proteins (SOCE) and TRPC channels to maintain the appropriate ER [Ca^2+^] that regulates neuronal functions (Sun et al., 2014). We found that dantrolene, a known blocker of the resting RyR-leak and RyRs-mediated Ca^2+^ release, reduced [Ca^2+^]_r_ and improved cognitive functions in middle-aged and aged-neurons. Dantrolene reduces intracellular [Ca^2+^] in the muscle (Lopez et al., 1987a,b, 1988) and neurons (Robin et al., 2017) and is currently used clinically to treat malignant hyperthermia (Harrison, 1988). Furthermore, dantrolene attenuates age-associated spatial memory deficits (Hopp et al., 2014) and has been previously shown to improve cognition deficits in a murine model of Alzheimer’s disease (Peng et al., 2012). Thus, the positive effects of dantrolene on [Ca^2+^]_r_ and cognitive functions in aged mice may be due to inhibition of excessive RyR-mediated Ca^2+^ leak.

### Critical Role of TRPCs During Aging

TRPC channels are widely expressed in almost every mammalian cell. TRPC channels can be activated by diverse stimuli ranging from temperature, mechanical or osmotic stress, chemical compounds, and redox modification (Sawamura et al., 2017; Samanta et al., 2018). Increased TRPC expression has been associated with aging in the heart and aorta (Kaplan et al., 2007; Erac et al., 2010), and with neurodegenerative diseases (Selvaraj et al., 2010; Takada et al., 2013; Sukumaran et al., 2017). In our studies looking into the mechanisms behind the changes in [Ca^2+^]_r_ in aging hippocampal neurons, we found that if we incubated them with the TRPC3/TRPC6 blocker SAR7334 (Maier et al., 2015), [Ca^2+^]_r_ and [Na^+^]_r_ were lowered in neurons in all three age groups suggesting that that increased plasmalemmal Ca^2+^ influx through TRPC channels was at least in part responsible for the increased [Ca^2+^]_r_ and that lowering the activity of these TRPC channels could protect the neurons from the detrimental effects of excessive intracellular [Ca^2+^] and [Na^+^]. Thus, TRPC channels not only contribute to normal physiological processes but are also implicated in aging. It suggests that these channels could be used as potential therapeutic targets to prevent the neuronal changes observed during aging.

### Calpain Activation and Cell Viability

Interestingly, the increased [Ca^2+^]_r_ in hippocampal neurons from middle-age and aged mice was associated with increased neuronal calpain activity, and reduced cell viability compared to young neurons. Calpain activation has previously been reported in aged neurons (Nixon, 2003) and proposed to be involved in age-associated neurodegenerative conditions such as Alzheimer’s disease (Battaglia et al., 2003; Nixon, 2003). Pretreatment with dantrolene, which reduces [Ca^2+^]_r_, lowered calpain activity to or near to young neuron levels and improved cell viability in both middle-aged and aged neurons. This demonstrates that dantrolene has a neuroprotective effect, which is at least in part if not completely mediated by its ability to reduce [Ca^2+^]_r_. This neuronal Ca^2+^ dyshomeostasis and decreased neuronal viability in middle-aged and aged mice was associated with significant age-related learning and cognitive memory deficits as measured by the MWM. As with the biochemical studies, these learning and memory deficits were significantly improved by dantrolene treatment.

### Cognitive Function, Ca^2+^ Overload, and Aging

Using the MWM test (Morris, 1984), we assessed the learning and memory skills of spatial position and direction in young, middle-aged and aged mice. Escape latency was used to evaluate learning ability and memory retention and the number of platform crossings was used to evaluate spatial memory ability. Unexpectedly we found that these cognitive functions had already declined in middle-age compared with young mice as well as the expected decline in aged mice. Age-related cognitive decline has been associated with alterations of intracellular [Ca^2+^]_r_ (Wu et al., 2002) and degenerative neurological pathologies including Alzheimer’s and Parkinson’s disease (Disterhoft et al., 1994; Kirischuk and Verkhratsky, 1996; Lopez et al., 2008). Based on the present results, it is appealing to suggest that a neuronal intracellular Ca^2+^ dysregulation such as that observed in middle-aged and aged neurons predisposes to the development of age-related cognitive decline in mice but perhaps also in the pathophysiology of this phenomenon in humans as well.

### Dantrolene Neuroprotection and Aging

Dantrolene is a hydantoin derivative which is currently the drug of choice for the treatment of patients suffering from malignant hyperthermia (Riazi et al., 2018). Dantrolene is also clinically useful for the treatment of symptoms associated with spasticity (Chou et al., 2004), and rhabdomyolysis induced by exercise (Edwards et al., 2003). Furthermore, dantrolene has been shown to have neuroprotective effects in multiple models of neurodegenerative disorders like Huntington’s disease (Chen et al., 2011) and Alzheimer’s disease (Wang et al., 2017; Liang and Wei, 2015). The muscle relaxant properties of dantrolene have been linked with its ability reduce [Ca^2+^]_r_ in humans (Lopez et al., 1992), swine (Lopez et al., 1987a) and mice (Yang et al., 2006; Cherednichenko et al., 2008; Eltit et al., 2013) although the exact mechanisms by which dantrolene exerts its therapeutic effects both in muscle and neurons are not fully understood. The predominant data supports the view that in skeletal muscle dantrolene acts selectively on the sarcoplasmic reticulum (SR) Ca^2+^ release channel (ryanodine receptor type 1, RyR1) to reduce SR leak and maintain normal SR Ca^2+^ stores (Fruen et al., 1997). In our model of normal aging, pretreatment with dantrolene appears to exert a neuroprotective effect on middle aged and aged neurons by reducing neuronal intracellular Ca^2+^ overload. When neuronal [Ca^2+^]_r_ is normalized elevated calpain activity, which is found in untreated neurons, is decreased and this decrease improves cell viability. In addition, we demonstrated that when dantrolene is given chronically for 10 days it is able to improve cognitive deficits observed in untreated middle aged and aged mice.

### Study Limitations

Despite the originality of our study, some limitations should be acknowledged. First, we explored intracellular Ca^2+^ dysregulation as the only factor contributing to the pathogenesis of aging and not other hypotheses such as: (i) glial cell alterations (Popa-Wagner et al., 2019), (ii) blood-brain-barrier disruption (Fulop et al., 2019), (iii) alteration in circadian rhythms (Logan et al., 2018), and (iv) endothelial dysfunction (Tarantini et al., 2019). Second, we did not perform a dose-response curve on dantrolene and cognitive improvement. Third, we did not assess whether blocking TRPC channels or genetic manipulation TRPC and RyR channels can rescue or improve cognitive deficit in aged-mice. Finally, we also acknowledge the small number of mice used for behavioral testing.

## Conclusions

In summary, the novel findings of the present study are: (i) the direct measurement of [Ca^2+^]_r_ in cortical neurons *in vivo* using Ca^2+^ selective microelectrodes; (ii) the demonstration of dysregulation of [Ca^2+^]_r_ in middle-aged as well as aged cortical and hippocampal neurons; (iii) the revelation that the elevation in [Ca^2+^]_r_ in middle-aged and aged neurons can be decreased or normalized by either dantrolene or SAR7374; (iv) that disruption of normal neuronal intracellular Ca^2+^ homeostasis leads to increases in calpain activity and a decrease in neuronal viability; (v) neuronal [Ca^2+^]_r_ dysregulation is associated with an age-related cognitive decline, as measured by MWM performance; and (vi) the reduction in [Ca^2+^]_r_ associated with dantrolene treatment in middle-aged and aged animals was accompanied with an improvement of cognition as measured by MWM.

Based on the above findings, it appears there is an interaction between cognitive decline observed in middle-aged and elderly mice and chronic intracellular Ca^2+^ dysregulation. Despite that alterations in ER-RyR Ca^2+^ leak and TRPC channels may not be the only pathogenic factors contributing to the pathogenesis of aging, the potential modification of those alterations is enough to enable expectations for new preventive or therapeutic strategies. Further research must be carried out to explore more in-depth the role of intracellular Ca^2+^ dysregulation and cognitive decline observed with aging.

## Data Availability Statement

The raw data supporting the conclusions of this article will be made available by the authors, without undue reservation, to any qualified researcher.

## Ethics Statement

All protocols used in the study were performed following the recommendations in the Guide for the Care and Use of Laboratory Animals of the National Institutes of Health and approved by the IACUC of the Mount Sinai Medical Center.

## Author Contributions

AU and VF: performed research and analyzed data. JA and PA: analyzed data and wrote the article. JL: designed research, performed research, analyzed data and wrote the article. All the authors contributed to manuscript revision, read and approved the submitted version.

## Conflict of Interest

The authors declare that the research was conducted in the absence of any commercial or financial relationships that could be construed as a potential conflict of interest.
